# 

**Published:** 1825-08

**Authors:** 


### MONTHLY CATALOGUE OF MEDICAL BOOKS.

*** *It having been hinted to us that gentlemen, sending copies of their Works, prefer having their titles given at length, it is our intention, in future, to comply with this suggestion: but it is to be observed, that no books can be entered on this List except those sent to us for the purpose;—finding, in the lists hitherto transmitted, that the names of works have frequently been given as published, which have not appeared for weeks, or even months, after.*

Military Medical Reports, containing Pathological and Practical Observations, illustrating the Diseases of Warm Climates. By JAMES M'CAVE, M.D. Author of Observations on the Cheltenham Waters, &c. &c.—Cheltenham, 1825.

A Course of Dissections for the Use of Students. By HERBERT MAYO, Surgeon and Lecturer on Anatomy.—London, 1825.

Practical Remarks upon Indigestion; particularly as connected with Bilious and Nervous Affections of the Head and other parts; including Observations upon the Disorders and Diseases of the Stomach and superior parts of the Alimentary Canal. Illustrated by Cases. By JOHN HOWSHIP, Assistant Surgeon to the St. George's Infirmary, &c. &c.—London, 1825.

Observations on Gout, Critical and Pathological; or, an Analytical Survey of the Views at present entertained of the Nature of that Disorder. With practical Remarks on the injurious Effects of Colchicum, and on certain Modes of Diet. By A. RENNIE, Surgeon.—London, 1825.

Medicinish-Chirurgische Zietung. Zweiter band.—Insprack, 1825.

Allgemeine Medizinische Annalen, February and March, 1825.—Leipzig.

### NOTICE TO CORRESPONDENTS.

*We beg to acknowledge the receipt of Communications from* Mr. Litchfield, Mr. Bush, Dr. Venables, Mr. Thomas, Mr. Chessher, *&c.*

Mr. Money's *Paper will certainly appear in our next Number.*

*We return our thanks to* Dr. Von Busch *for his enclosures, and the handsome letter which accompanied them.*

